# Longitudinal dynamics of the bovine udder microbiota

**DOI:** 10.1186/s42523-022-00177-w

**Published:** 2022-04-08

**Authors:** Anja Ruud Winther, Judith A. Narvhus, Marit Smistad, Vinicius da Silva Duarte, Alberto Bombelli, Davide Porcellato

**Affiliations:** 1 Faculty of Chemistry, Biotechnology and Food Science, The Norwegian University of Life Sciences, Ås, Norway; 2 Norwegian Veterinary Institute, Oslo, Norway; 3 TINE SA, Oslo, Norway; 4 Department of Agrotechnology and Food Science, Wageningen University and Research, Wageningen, Netherlands

**Keywords:** Microbiota, Bovine milk, Temporal dynamics

## Abstract

**Background:**

In recent years, the number of studies concerning microbiota of the intramammary environment has increased rapidly due to the development of high-throughput sequencing technologies that allow mapping of microbiota without culturing. This has revealed that an environment previously thought to be sterile in fact harbours a microbial community. Since this discovery, many studies have investigated the microbiota of different parts of the udder in various conditions. However, few studies have followed the changes that occur in the udder microbiota over time. In this study, the temporal dynamics of the udder microbiota of 10 cows, five with a low somatic cell count (SCC, SCC < 100,000 cells/mL) and five with a high SCC (SCC > 100,000 cells/mL), were followed over 5 months to gather insights into this knowledge gap.

**Results:**

Analysis of the temporal changes in the microbial composition of milk from udders with a low SCC revealed a dynamic and diverse microbiota. When an imbalance due to one dominating genus was recorded, the dominant genus quickly vanished, and the high diversity was restored. The genera dominating in the samples with a high SCC remained the dominant genera throughout the whole sampling period. These cows generally displayed a heightened SCC or an intramammary infection in at least one quarter though-out the sampling period.

**Conclusion:**

Our results show that the bovine udder has a diverse microbiota, and that the composition and diversity of this community affects udder health with regards to SCC. Understanding what influences the composition and stability of this community has important implications for the understanding, control, and treatment of mastitis.

**Supplementary Information:**

The online version contains supplementary material available at 10.1186/s42523-022-00177-w.

## Background

The microbiota of the bovine udder is receiving renewed interest due to the use of sequencing methods which appear to give more detailed information than culture-based methods about the microorganisms which colonize intramammary tissues. Many reports of udder microbiota are compromised by challenges of avoiding contamination from the teat apex and cisterns during sampling [1, 2]. Since the bacterial load in the healthy udder is low, contamination will always be a potential problem with samples collected by expressing milk from the teat orifice. Only when an intramammary infection is present is the bacterial population large and is therefore easy to detect. This has led to the supposition that the healthy udder is sterile, and that bacteria are only present during an infection [3]. However, increasing knowledge of the importance of the microbiota of all organs has shown that organs previously thought to be sterile do in fact have their own microbiota [2, 4–6]. The composition of the commensal udder microbiota can be confounded by sampling technique and is influenced by a number of factors, e.g. housing, management practices and type of bedding material, which make comparisons between studies difficult [7, 8]. The microbiota of bovine milk is highly diverse and typically includes taxa from the phyla *Firmicutes, Proteobacteria, Actinobacteria*, and *Bacteroidetes*, with the most common genera being *Staphylococcus*, *Streptococcus*, *Ruminococcaceae*, *Lachnospiraceae*, *Propionibacterium*, *Stenotrophomonas*, *Corynebacterium*, *Pseudomonas*, *Fusobacterium*, *Lactobacillus*, *Enterococcus*, *Comamonas*, and *Bacteroides* [1]. The commensal species found on the external teat skin, in the teat canal and in the udder lumen might function as important protective elements against pathogens, as is shown for commensal species of other body sites [1, 9, 10]. Studies show that certain species of non-aureus *Staphylococci* and *Corynebacterium* produce bacteriocins that prevent growth of potential pathogens and are consequently hypothesized to be involved in protecting the bovine udder against mastitis [11, 12].

Despite this, pathogens are able to breach the udder defences causing intramammary infections (IMI) that can result in mastitis [13]. When comparing the microbial composition of healthy and mastitic milk samples, a shift in the population is typically detected where the infectious genus becomes dominating. This leads to the reduced diversity often seen in mastitic milk samples [14, 15]. An open question is whether this imbalance causes mastitis, or vice versa. Moreover, some studies have revealed that genera considered to cause mastitis can be part of the commensal udder microbiota [2, 15]. An event that would grant this subpopulation an advantage over the other commensal species would allow rapid growth of the pathogen, causing imbalance in the population [1]. On the other hand, when a pathogenic bacterium is cleared from the udder by the immune system, the imbalance caused by this species would likely persist until the commensal bacteria that were repressed by the pathogen are able to grow back to the original population size [16, 17]. Whether the commensal species are repressed when a pathogen is present, or if the pathogen is so dominating that the commensal species are not detected during sequencing is unknown.

A common practice to characterize the health status of cows is measuring the number of somatic cells in the milk. The SCC in the lumen of an infected quarter increase due to migration of white blood cells to the site of infection and increased shedding of the epithelial cells from the lumen wall [18]. In milk from a healthy udder, SCC levels are from 10–50,000 cells/mL and in cases of clinical mastitis, the levels are likely to be over 1 million cells/mL. Subclinical mastitis shows a broad range, between 100,000 and several million cells/mL. The allowable levels in milk for delivery to the dairy vary in different countries, but under 400,000 cells/mL as a rolling mean over three months is often used [19]. Using this level, it is likely that a number of individual cows in the herd have subclinical mastitis. In addition, if the best relationship between SCC and IMI is seen at the quarter level, then pooled milk from one cow may not show very high levels if only one quarter is infected [20].

Most published studies regarding the microbiota in bovine milk samples represent a small part of the actual picture as they often analyze samples taken at a single timepoint [4, 15, 21]. Only a few publications have addressed potential temporal changes of the udder microbiota [2, 16, 22]. In this study, we aimed to increase knowledge regarding these temporal dynamics. We collected quarter samples from 10 cows every three to four weeks over five months. The cows were chosen based on their SCC in the days leading up to the first sampling. Five cows were included in the study due to their low SCC indicating a healthy udder, while the remaining five were included for their high SCC, which would be consistent with an imbalance of the microbiota of the udder and possibly subclinical mastitis. Amplicon sequencing of the 16S rRNA gene was used to analyze the samples. This revealed that the healthy quarter contains a dynamic bacterial population that changes with time, while the imbalanced quarter has a lower diversity bacterial population dominated by a single genus.

## Results

### General characteristics of the cows and quarter samples included in the study

This study aimed to investigate the temporal dynamics of the microbiota in the bovine udder. The ten cows used for this purpose were divided in two groups based on the level of SCC before the first sampling. Five of the cows (L1–L5) had a stable low SCC (< 100,000 SCC/mL) on the three days leading up to the first sampling, while the remaining five cows (H1–H5) had a higher SCC (> 100,000 SCC/mL) in the same period. Quarter milk samples were collected from all ten cows at six samplings during a period of five months (January to May). Of the 240 quarter samples that were collected, six were missing during collection and were not included in the analysis. To study the microbial composition of the 234 remaining samples, amplicon sequencing of the 16S rRNA genes were performed for all the samples. The average depth of sequencing was 49,093 sequences per sample before filtering and 18,880 sequences per sample after filtering. In total 9132 high quality SVs were obtained from 234 samples. 14 samples were filtered out of the analysis because they did not pass the quality filtering of the Dada2 pipeline used to analyse the 16S data after sequencing. Of the 9,132 high quality SVs, 6962 SVs were used for taxonomy search, and 553 SVs were successfully assigned to family level. 61 quarter samples were classified as having an IMI based on definition “A” from Dohoo et al. [23]. This definition states that a quarter sample where > 10 colonies are cultured per 0.1 mL is defined as having an IMI. Eighty-nine percent of these were from group H and 11% were from group L. A limit of 100,000 SCC/mL was selected to classify the quarter samples as high or low SCC during samplings. 30 quarter samples had high SCC, 204 low SCC, and 6 samples had no recorded SCC. Of the 30 samples with a recorded high SCC, 93% were from group H and 7% from group L. Other additional data about the health, parturition, and recorded mastitis were retrieved from the Norwegian Cattle Health Recording System (Additional file 1). No cows were recorded to have mastitis during the sampling period, while two cows (L1 and H2) were treated for mastitis caused by *Streptococcus dysgalactiae* and *Streptococcus uberis*, respectively, after this period. The samplings occurred between 19 and 193 days in milk for the 10 cows (Additional file 2). This period encompasses the early and mid-lactation stages. Five of the cows (H4, H5, L3, L4, L5) were in their first parturition, while the remaining five (H1, H2, H3, L1, L2) were in their second parturition.

### Diversity analysis of the quarter samples

**Fig. 1:**
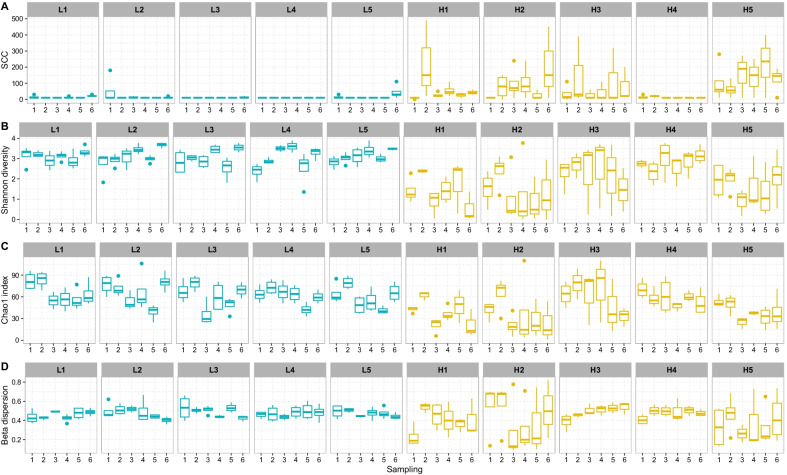
(**A**) SCC, (**B**) Shannon diversity, (**C**) Chao1 index, and (**D**) Multivariate homogeneity of group dispersions. **A**, **B**, **C**, and **D** are plotted per individual cow (blue represents L-cows, yellow represents H-cows) and shown for all six samplings

Investigation of the alpha diversity shows that the cows with a low SCC before sampling, hereafter referred to as L-cows, displayed a stable and more diverse microbiota in the udder compared to the cows with a high SCC before sampling (H-cows). The SCC was plotted for each individual cow and shown for all six samplings in Fig. 1A. The diversity was measured both as species relative abundance with a focus on evenness and dominance (Shannon diversity, Fig. 1B) and species richness estimation (Chao1 index, Fig. 1C), with an average Shannon diversity of 3.08 and Chao1 index of 62.42 for the L-cows. The H-cows had an average Shannon diversity of 1.88 and Chao1 index of 46.25. Statistical testing with Kruskal–Wallis rank sum test confirmed that the observed difference between the two groups was significant (*p* < 0.05). The quarter samples characterized as having an IMI and the samples characterized as having a high SCC during sampling also had a species abundance estimate and species richness estimate that were significantly different from the samples without IMI and with a low SCC, respectively (*p* < 0.05). The generally larger spread of multivariate dispersion recorded for the H cows (Fig. 1D) indicates a less stable microbiota in the udder of these cows.

**Fig. 2:**
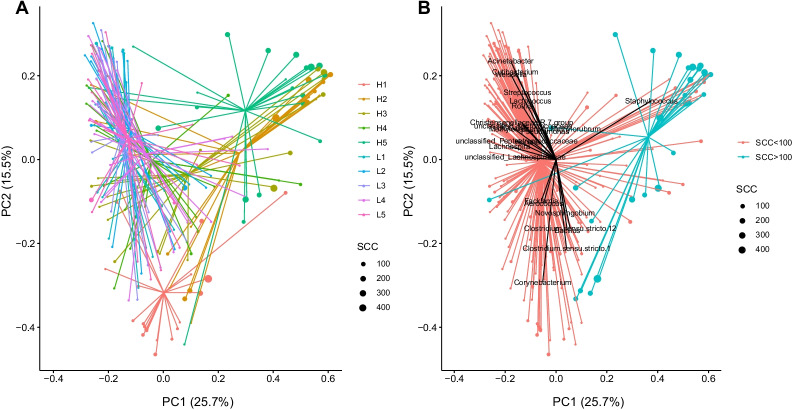
PCoA of the udder milk samples obtained from 10 cows over 5 months of sampling and grouped by **A** cow ID, and **B** SCC during sampling. Size of the points indicate the level of SCC

Beta diversity analyses confirmed the observed difference between the two groups of cows and between the samplings. The permutational multivariate analysis of variance using distance matrices showed significantly different microbial composition (Adonis *p* < 0.001) between the H-cows and L-cows and between samplings. The interactions between the two factors (H/L-cows and the samplings) also obtain a *p* value < 0.001. Principal coordinates analysis (PCoA) based on the Bray–Curtis dissimilarity matrix as input was employed to investigate diversity between the samples. The scatterplot in Fig. 2A displays a clear difference between the H- and L-cows. The L-cows grouped similarly to each other (on the left side of the plot), while the H-cows were less similar to each other and to the L-cows. One exception is H4 which is found grouped with the L-cows. This is also reflected in Fig. 1A (Shannon diversity), where H4 displays a higher diversity compared to the rest of the H-group. A distinct difference between the clustering of groups can also be seen when grouping the samples based on SCC during samplings (Fig. 2B).

### Differential abundance analysis of significant taxa

**Fig. 3:**
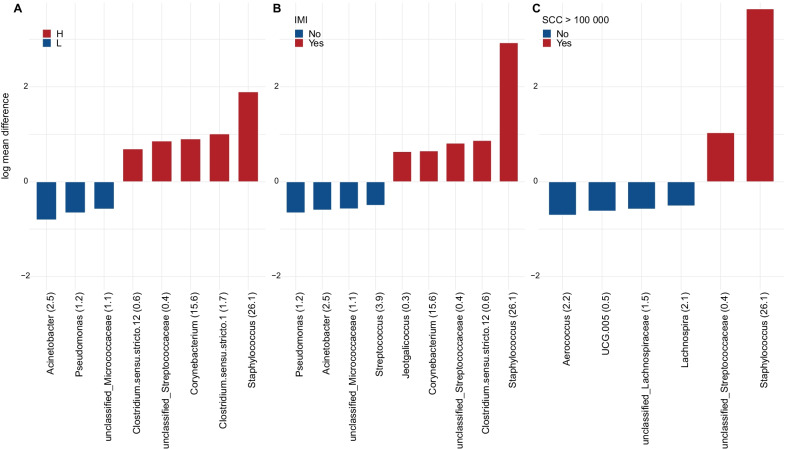
Differential abundance of the statistically significant general (*p* < 0.05) plotted for **A** H- and L-cows, **B** presence of intramammary infection (according to definition “**A**” by Dohoo et al. [23]) and **C** somatic cell count (lower or greater than 100,000 SCC/mL) during sampling. Numbers in parenthesis after the genus names indicate the genus percentage of total reads

Identification of genera significantly more abundant in quarters within the different groups was performed with Analysis of Compositions of Microbiomes with Bias Correction (ANCOM-BC). The three conditions “H-cows”, IMI positive and SCC > 100,000/mL (Fig. 3A–C, respectively) were associated with Gram-positive genera such as *Staphylococcus* and the family *Streptococcaceae*. This is in accordance with the PCoA plot in Fig. 2B where *Staphylococcus* associates with the samples with a raised SCC. On the contrary, the microbiota of the healthy conditions “L-cows”, IMI negative and SCC < 100,000/mL had a balanced composition of Gram-positive and Gram-negative genera.

### Analysis of the most abundant taxa present in the quarter samples

**Fig. 4:**
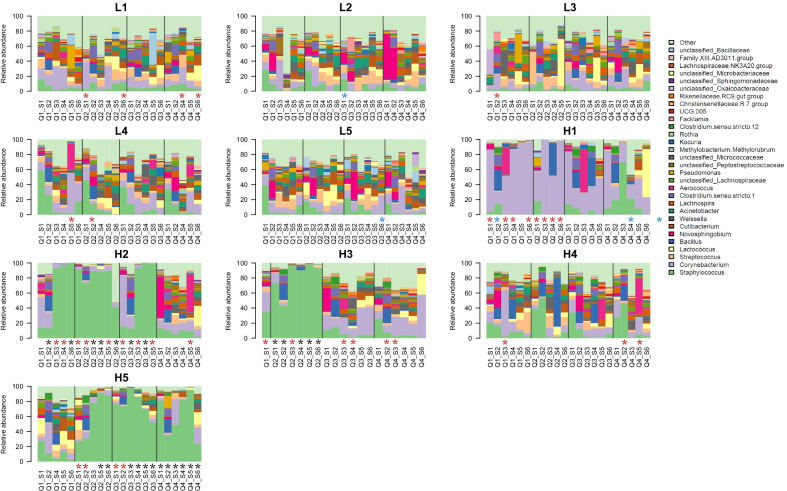
The relative abundance of the 30 most abundant genera plotted for each quarter sample and displayed by cow ID over time. Each bar indicates a quarter sample, where Q1–4 represents the quarter (Q1: right back, Q2: left back, Q3: right front, Q4: left front) and S1–6 represents the sampling number. The asterisks indicate samples with recorded IMI (red, > 10 cultured colonies per 0.1 mL), SCC > 100,000/mL (blue) or both (black)

A total of 553 sequence variants were successfully assigned to the family level and kept for further analysis. The relative abundance of the 30 most abundant genera is displayed by quarter sample in Fig. 4. Fifty-seven percent of these belong to the phylum Firmicutes, 20% to Actinobacteria, 20% to Proteobacteria, and the remaining three percentages belong to the Bacteroidetes. The most abundant genera were *Staphylococcus* and *Corynebacterium* with 26.1% and 15.6% of the total sequences assigned to family level in this study, respectively. Figure 4 shows that the L-cows presented a diverse microbiota composition in all quarters. As opposed to the H-cows that typically displayed one or more quarters where one genus was dominating, the L-cows had a richer diversity with several genera present simultaneously. We also observed large differences between quarter samples from the same cow at the same sampling. As earlier mentioned, and as evident by the bar plots in Fig. 4, the H-cows typically had one or two quarters with low diversity where one genus dominated. Interestingly, the dominant genera causing the reduced diversity were *Staphylococcus *and *Corynebacterium*. These were also present in the L-cows but did not seem to be able to dominate the microbiota in these quarters. The quarter samples recorded to have IMI, SCC > 100,000/mL, or both are labelled with an asterisk in Fig. 4 (red, blue or black, respectively). The two conditions recurred mainly in the H-cows. As observed when investigating the alpha- and beta-diversities, cow H4 was more similar to the L-cows regarding microbial composition.

### Temporal changes of the microbial community in the bovine udder

Temporal changes in relative abundance of the main genera present in the udder microbiota were detected for all the cows (Fig. 4). We saw a flare of *Novosphingobium* in both cows L2 and L5 during the first and second sampling that were not present in the subsequent months. *Lactococcus*, barely present in cow L3 during the early samplings, appears during the fifth sampling. This argues for a highly dynamic udder microbiota. On the other hand, *Staphylococcus* got a foothold in one quarter in cow H3 and remained thought-out the whole sampling period, suggesting that once this bacterium has established dominance in the quarter, it is difficult for the cow to get rid of again. This was also the case for the rest of the H-cows: once *Staphylococcus* (H2, H3, H5) or *Corynebacterium* (H1) had taken over in the udder, they remained the dominant genera over time, implying that re-establishment of a diverse community, as seen in healthy cows, is difficult. The difference in development of IMI in cows H2 and H3 is also worth mentioning. Both cows started the sampling period with two infected quarters but cow H2 developed infection in all quarters as time went by, while cow H3 mainly had one, at times two, infected quarters. Considering the infection status of the H-cows, it is interesting that when one quarter of cow L4 was overtaken by *Corynebacterium* and *Novosphingobium* in the fourth and fifth samplings, the diverse microbiota typical for L-cows had been reestablished by the sixth sampling. This flare of *Corynebacterium* and *Novosphingobium* lead to an IMI (labeled with red asterisk in Fig. 4) as seen in the H-cows when a genus takes over a quarter. The difference is that in the imbalanced quarter of cow L4, the issue was resolved spontaneously, while in the H-cows, the imbalance persists.

## Discussion

Increased knowledge of the udder microbiota is an important step in understanding mastitis dynamics, a disease affecting herd health and milk production yields world-wide [1]. To date, however, only a few studies have used HTS technologies to determine longitudinal shifts in the bacterial community of milk samples collected from healthy quarters [16, 22]. In the present study, we aimed to investigate the temporal changes in the udder microbiota of Norwegian Red cows over 5 months, which encompassed both the early and mid-lactation stages. The bacteria present were identified through amplicon sequencing of the V3-V4 region of the 16S rRNA genes. During library preparation, amplification in the negative controls were not detected in the qPCR system and it is hence unlikely that the samples suffer from contamination. For that reason, the controls were not included in the data analysis. In an attempt to acquire bacteria from deep within the udder, samples were collected after regular milking. This practice was used to avoid contamination of bacteria from the environment that had entered the teat apex [2, 3, 21]. Without invasive methods it is not possible to rid the samples of all contaminants, but Porcellato et al. [2] employed this type of sampling technique, and despite finding evidence of some environmental genera in the samples, their relative abundance was lower compared to milk taken from bulk milk tanks. This suggests that employing this sampling technique rids the samples of some of the environmental bacteria that are present in the teat apex. Three to four weeks were chosen as an interval between sample collection. This means that a transient subclinical intramammary infection could be missed as the pathogen could be cleared from the udder before the next sampling. However, no case of mastitis or mastitis treatment was recorded in the Norwegian Cattle Health Recording System through the duration of the experiment for the cows in the study.

The detection of *Staphylococcus* or *Corynebacterium* in all quarter samples were consistent with other studies [2, 14, 15, 22]. The udder microbiota of the L-cows turned out to be highly dynamic and diverse with fluctuations between the relative abundance of genera from one sampling to the next. In the one case where an IMI was detected and accompanied by an increase in the relative abundance of *Corynebacterium* and *Novosphingobium*, a diverse microbiota composition had been reestablished by the next sampling. A different tendency was recorded for the H-cows. In quarters with an established IMI or a raised SCC due to dominance of one genus (*Staphylococcus* or *Corynebacterium*), returning to a diverse microbiota composition turned out to be difficult, and the dominant genus was found to linger throughout the whole sampling period. Some quarters appear to be dominated by *Corynebacterium* or *Staphylococcus,* yet they were culture negative under both aerobic and anaerobic conditions and have therefore not been labeled as having an IMI. This inconsistency between the 16S data and the culturing results is likely due to the inability of laboratory culturing methods to cultivate all bacteria in a sample. The criteria used to define a quarter sample as IMI positive in this study was chosen to identify as many IMIs as possible. Whilst definition “A” from Dohoo et al. [23] certainly achieves this, it can also introduce false positives to the dataset. To make up for this it would have been prudent to include species identification after plating on blood agar as an additional screening method for quarters with IMI.

Several factors might be responsible for the development of different microbiota between the H- and L-cows. It is possible that *Corynebacterium* or *Staphylococcus* were able to settle deep inside the udder of certain quarters of the H-cows. If that is the case, they could persist here until conditions were favorable for proliferation. As described elsewhere, some mastitis pathogens can colonize distinct anatomical niches of the udder for long periods by expressing virulence factors impairing the total or partial reestablishment of the resident microbiota [1, 24]. Indeed, it has been observed that this feature might be strain-specific as has been observed for naturally or experimentally infected quarters with *E. coli* where a normal udder microbiome was promptly restored following pathogen clearance within the mammary gland [16, 17]. Isaac et al. [25] found that non-pathogenic coagulase-negative *Staphylococcus chromogenes* from bovine milk samples produced an anti-biofilm compound that hinders the establishment of pathogenic bacteria in the udder. Al-Qumber et al. [26] published similar results regarding *Bacillus* isolated from healthy quarters. These protective commensal species may explain why *Corynebacterium* and *Novosphingobium* could not establish a foothold in cow L4. The interplay between the commensal microbiota and potential pathogens can also explain how cow H3 was able to contain an IMI in one quarter, while H2 developed IMI in several quarters during the sampling period. It is possible that the uninfected quarters harbor species as part of their microbiota that are able to repress growth of the opportunists, while these species might be lacking or present in too few numbers in the quarters that are more susceptible to developing an IMI. Another known factor that increases the occurrence of mastitis is an increasing number of parturitions, and the likelihood of mastitis is also higher in the periparturient period [21, 27, 28]. Due to the number of cows included in this study, we do not have sufficient data to conclude anything regarding this claim. Another factor contributing to mastitis susceptibility might be the genetic composition of the cow, particularly the genes encoding the bovine leukocyte antigens, and factors influencing general host resistance, such as stress and nutrition. [29]. These factors have not been evaluated in this study.

Milk samples collected from cows with SCC < 100,000/mL and without IMI showed a stable eubiotic bacterial community throughout samplings as evidenced by higher species abundance and species richness estimates, which is commonly observed for milk samples obtained from healthy udders [4, 15, 17]. Interestingly, it is possible to observe that samples classified as “H”, IMI positive, and SCC > 100,000/mL are remarkably enriched by Gram-positive genera (i.e., *Staphylococcus*, *Clostridium*, *Corynebacterium*, *Jeotgalicoccus*, and *Streptococcus*), whereas milk samples from healthy quarters display a balance between Gram-positive and Gram-negative taxa. It is worth mentioning that Gram-negative genera such as *Pseudomonas* and *Acinetobacter* occur mainly in milk samples collected during the pasture season [1]. Although it is difficult to establish whether the low diversity found in milk samples from H-cows is a cause or a consequence of ongoing subclinical mastitis, our results reinforce the association of mastitis with an imbalanced udder microbiota [2].

Regarding the temporal changes in the relative abundance of the main bacterial genera present in the udder microbiota of H- and L-cows, *Pseudomonas* are overrepresented in the microbiota of healthy quarters [14], whereas *Acinetobacter*, *Aerococcus*, and *Corynebacterium* are among the most frequently identified genera on the skin of teat apices [1, 30]. According to Falentin et al. [27], *Jeotgalicoccus* and *Corynebacterium* were more abundant in foremilk samples of quarters with a history of mastitis, an important bacterial source involved in udder’s colonization and IMI. Indeed, in a study conducted with milk and teat skin samples from 1142 quarters (300 cows with somatic cell count > 200,000 cells/mL), Svennesen et al. [31] indicate that *S. aureus* and *S. agalactiae* present on teat skin can be considered a risk factor for IMI caused by these species. The family *Staphylococcaceae* include Gram-positive spherical bacterial species of veterinary interest such as *S. aureus* and non-*aureus* staphylococci (NAS). These are some of the most common mastitis related pathogens, and an intramammary infection caused by *S. aureus* before peak lactation can lead to a milk loss of up to 7.1% [32]. NAS species and *Corynebacterium bovis*, a minor mastitis causing pathogen, also leads to loss in milk production, 7.4% and 5.7%, respectively. Together, our results and the aforementioned studies demonstrate the importance of a routine of monitoring and identification of animals with intramammary infection within the mastitis control program, i.e., monitoring of SCC and bacterial counts. Lastly, the gut-associated families *Clostridiaceae* and *Lachnospiraceae* are frequently reported as members of the teat canal microbiota when cows were housed in free or tie-stall arrangements [1], although Andrews et al. [22] have identified a prevalence of both families in teat samples of lactating organic dairy cows (hay and pasture-based system) regardless of infection status, which evidences the factor “environment” in shaping the udder microbiota.

## Conclusion

This study contributes new knowledge about temporal changes of the udder microbiota and is important for understanding udder health and the development of mastitis. The results indicate that it could be possible to identify the risk of developing IMI in a quarter based on the composition of the microbial community present, such as diversity and balance between Gram-positive and Gram-negative species. We showed that different udder microbiomes behave differently depending on udder health. A highly diverse microbiota was more stable over time, subject to less disturbance and associated with healthier udders. This highly diverse microbiota displayed a balance between Gram-positive and Gram-negative species not detected in udder microbiotas with lower diversity. The low diversity microbiota was subjected to temporal shift in composition and was correlated to infected quarters and the presence of mastitis pathogen genera. The latter microbiota was generally dominated by Gram-positive species.

## Materials and methods

### Study animals

Ten Norwegian Red cows were selected from the “Centre for livestock production” at the Norwegian University of Life Sciences. The farm operates under the regulations of the Norwegian Food Safety Authority regarding food production and animal care. Permission for sample collection and use of information regarding the samples was given by the farm owners. No invasive procedures were used in this study. The cattle enrolled in the study were housed in freestalls with cubicles containing bedding materials of rubber mats with raw wood chips. Their diet consisted of silage, continuously available, and supplemented with pelleted feed based on milk production of the individual cow. The ten cows included in the study were chosen based on SCC recorded by the automatic milking system (Delaval Online Cellcounter) in the three days leading up to the first sampling. Five cows with a constant low SCC (< 100,000/mL, L1–L5) and five cows with a constant high SCC (> 100,000/mL, H1–H5) were selected for the study. The Norwegian Cattle Health Recording System provided additional metadata for each of the cows [33] (Additional file 1).

### Sample collection

Milk samples were collected from each quarter on six occasions over 5 months with 3–4 weeks intervals between samplings (January–May 2020). Milk was collected at the end of the regular milking routine, as previously described by Porcellato et al. [2]. Briefly, after removal of the milking apparatus, the teats were washed with iodine and then alcohol, and 200 ml milk was collected manually. The “Procedure for Collecting Milk Samples” of the National Mastitis Council (NMC, www.nmconline.org) was followed. 240 samples were collected for the study. After sample collection, the milk was stored on ice until arrival in the laboratory (no more than 2 h after the last sample was taken) where the samples were immediately prepared for analysis. 100 µl of raw milk were plated on TSA blood agar plates (ThermoFischer Scientific, Massachusetts, United States). The agar plates were incubated at 37 °C under both aerobic and anaerobic conditions for 24 h. Airtight containers and AnaeroGen 3.5L sachets (ThermoFischer Scientific) were used to create anaerobic conditions. In order to identify quarters with IMIs, we utilized the definition “A” from Dohoo et al. [23]. Quarters were labelled with an IMI if they had > 10 colonies per 0.1 mL (pure or mixed culture) in one of the two conditions used for TSA blood agar (aerobic, anaerobic). Milk from all quarter samples were also sent to the Tine Laboratory in Heimdal and analyzed with Bentley FTS (Bentley Instrument Inc, Chaska, MN, USA) for somatic cells, fat, protein, lactose, urea, and FFA content (Additional file 1).

### DNA extraction and amplicon sequencing

For the analysis of the microbiota, the bacterial pellet was obtained from 40 mL of each quarter milk sample, as previously described [2]. Briefly, 40 mL of milk was centrifuged at 8000× *g* for 10 min, the fat layer removed with a sterile cotton swab, and the supernatant removed. The pellet was then washed twice with 2% citrate water, and DNA was extracted from each pellet using the DNeasy PowerFood Microbial Kit (Qiagen, Düsseldorf, Germany) starting from step 3 in the detailed protocol of DNeasy Powerfood Microbial Kit Handbook. For increased efficiency of lysis of difficult species additional 5 min of vortex time were added to step 6, bringing the total vortex time to 15 min. DNA was finally eluted in 50 µl elution buffer before storage at − 20 °C. Library preparation for amplicon sequencing using the Illumina Miseq platform was performed as described previously [2]. The V3 and V4 region of the 16S rRNA was amplified using the primers Uni340F (CCTACGGGRBGCASCAG) and Bac806R (GGACTACYVGGGTATCTAAT). PCR reagents and conditions were identical to the one described by Porcellato et al. [2]. Negative controls were included to monitor for contamination during DNA extraction and during library preparation. The final library concentration was then measured using Qubit 2 with the dsDNA HS kit (ThermoFischer Scientific) and quantitated using the KAPA Library Quantification kit (Illumina) before being sequenced on an Illumina MiSeq platform (Illumina) using the 2 × 300 bp V3 kit (Illumina).

### Sequence data analysis and statistical testing

Reads were quality filtered and trimmed using the Dada2 package using truncating of forward reads set to 260 bases and truncating of reverse reads set to 240 bases [34]. The error model in Dada2 was created using 1 million random filtered reads. Sequence variants (SV) was inferred using the Dada2 algorithm and removal of chimeras was performed using the function “removeBimeraDenovo” in the Dada2 R package. Sequence variants shorter than 375 base pairs were removed from the final table. Taxonomy was assigned using the Decipher R package [35] against the SILVA SSU database [36]. Samples with less than 1000 reads and SVs with less than 10 sequences were removed from the table.

All statistical analyses were performed using the R software [37]. Chao1 estimate and Shannon diversity were chosen to evaluate the alpha diversity and were calculated using the R package Vegan [38]. Pairwise comparison of the alpha diversity indexes between group levels was performed using the Kruskal–Wallis rank sum test. Multivariate homogeneity of group dispersions was calculated using the function “betadisper” available in the R package Vegan. Permutational analysis using dissimilarity matrix (“adonis” function from the R package Vegan) was used to test differences in the composition of the community between groups of samples (n. of permutation 999). Bray–Curtis dissimilarity matrixes were selected as input for ordination analysis using principal coordinate analysis (PCoA). ANCOM-BC was used to identify families that were significantly more abundant among groups of samples [39].

## Supplementary Information

**Additional file 1.** Metadata provided by the Norwegian Cattle Health Recording System.

**Additional file 2.** Day in milking for the 10 cows over the six samplings.

## Data Availability

The dataset supporting the conclusions of this article is available in the EBI repository, PRJEB47169, https://www.ebi.ac.uk/. Metadata is available as Additional file 1.

## Abbreviations

IMI: Intramammary infection
SCC: Somatic cell count
PCoA: Principal coordinates analysis
HTS: High throughput sequencing
ANCOM-BC: Compositions of microbiomes with bias correction

## References

[1] Derakhshani H, Fehr KB, Sepehri S, Francoz D, De Buck J, Barkema HW, Plaizier JC, Khafipour E (2018). Invited review: microbiota of the bovine udder: contributing factors and potential implications for udder health and mastitis susceptibility. J Dairy Sci.

[2] Porcellato D, Meisal R, Bombelli A, Narvhus JA (2020). A core microbiota dominates a rich microbial diversity in the bovine udder and may indicate presence of dysbiosis. Sci Rep.

[3] Rainard P (2017). Mammary microbiota of dairy ruminants: fact or fiction?. Vet Res.

[4] Oikonomou G, Machado VS, Santisteban C, Schukken YH, Bicalho RC. Microbial diversity of bovine mastitic milk as described by pyrosequencing of metagenomic 16s rDNA. 2012. 10.1371/journal.pone.0047671 PMC3474744 23082192

[5] Dickson RP, Huffnagle GB (2015). The lung microbiome: new principles for respiratory bacteriology in health and disease. PLoS Pathog.

[6] Moossavi S, Azad MB (2020). Origins of human milk microbiota: new evidence and arising questions. Gut Microbes.

[7] Doyle CJ, Gleeson D, O'Toole PW, Cotter PD (2017). Impacts of seasonal housing and teat preparation on raw milk microbiota: a high-throughput sequencing study. Appl Environ Microbiol.

[8] Metzger S, Hernandez L, Skarlupka J, Suen G, Walker T, Ruegg P (2018). Influence of sampling technique and bedding type on the milk microbiota: results of a pilot study. J Dairy Sci.

[9] Adnane M, Chapwanya A (2022). A Review of the diversity of the genital tract microbiome and implications for fertility of cattle. Animals.

[10] Bomar L, Brugger SD, Yost BH, Davies SS, Lemon KP. *Corynebacterium* accolens releases antipneumococcal free fatty acids from human nostril and skin surface triacylglycerols. mBio 7(1):e01725-15. 10.1128/mBio.01725-15 PMC4725001 26733066

[11] Braem G, Stijlemans B, Van Haken W, De Vliegher S, De Vuyst L, Leroy F (2014). Antibacterial activities of coagulase-negative staphylococci from bovine teat apex skin and their inhibitory effect on mastitis-related pathogens. J Appl Microbiol.

[12] Woodward W, Besser T, Ward A, Corbeil L (1987). In vitro growth inhibition of mastitis pathogens by bovine teat skin normal flora. Can J Vet Res.

[13] Paulrud C (2005). Basic concepts of the bovine teat canal. Vet Res Commun.

[14] Kuehn JS, Gorden PJ, Munro D, Rong R, Dong Q, Plummer PJ, Wang C, Phillips GJ (2013). Bacterial community profiling of milk samples as a means to understand culture-negative bovine clinical mastitis. PLoS ONE.

[15] Oikonomou G, Bicalho ML, Meira E, Rossi RE, Foditsch C, Machado VS, Teixeira AGV, Santisteban C, Schukken YH, Bicalho RC (2014). Microbiota of cow’s milk; distinguishing healthy, sub-clinically and clinically diseased quarters. PLoS ONE.

[16] Ganda EK, Bisinotto RS, Lima SF, Kronauer K, Decter DH, Oikonomou G, Schukken YH, Bicalho RC (2016). Longitudinal metagenomic profiling of bovine milk to assess the impact of intramammary treatment using a third-generation cephalosporin. Sci Rep.

[17] Ganda EK, Gaeta N, Sipka A, Pomeroy B, Oikonomou G, Schukken YH, Bicalho RC (2017). Normal milk microbiome is reestablished following experimental infection with *Escherichia coli* independent of intramammary antibiotic treatment with a third-generation cephalosporin in bovines. Microbiome.

[18] Wang N, Zhou C, Basang W, Zhu Y, Wang X, Li C, Chen L, Zhou X. Mechanisms by which mastitis affects reproduction in dairy cow: a review. Reprod Domest Anim. 2021. 10.1111/rda.13953 34008236

[19] Sargeant JM, Schukken YH, Leslie KE (1998). Ontario bulk milk somatic cell count reduction program: progress and outlook. J Dairy Sci.

[20] Schukken YH, Wilson DJ, Welcome F, Garrison-Tikofsky L, Gonzalez RN (2003). Monitoring udder health and milk quality using somatic cell counts. Vet Res.

[21] Taponen S, McGuinness D, Hiitiö H, Simojoki H, Zadoks R, Pyörälä S (2019). Bovine milk microbiome: a more complex issue than expected. Vet Res.

[22] Andrews T, Neher DA, Weicht TR, Barlow JW (2019). Mammary microbiome of lactating organic dairy cows varies by time, tissue site, and infection status. PLoS ONE.

[23] Dohoo I, Smith J, Andersen S, Kelton D, Godden S, Workers’Conference MR. Diagnosing intramammary infections: Evaluation of definitions based on a single milk sample. J Dairy Sci 2011;94:250–61. 10.3168/jds.2010-3559 21183035

[24] Pedersen RR, Krömker V, Bjarnsholt T, Dahl-Pedersen K, Buhl R, Jørgensen E. Biofilm research in Bovine Mastitis. Front Vet Sci 2021;8. 10.3389/fvets.2021.656810 PMC8138050 34026893

[25] Isaac P, Bohl LP, Breser ML, Orellano MS, Conesa A, Ferrero MA, Porporatto C (2017). Commensal coagulase-negative *Staphylococcus* from the udder of healthy cows inhibits biofilm formation of mastitis-related pathogens. Vet Microbiol.

[26] Al-Qumber M, Tagg J (2006). Commensal bacilli inhibitory to mastitis pathogens isolated from the udder microbiota of healthy cows. J Appl Microbiol.

[27] Falentin H, Rault L, Nicolas A, Bouchard DS, Lassalas J, Lamberton P, Aubry J-M, Marnet P-G, Le Loir Y, Even S (2016). Bovine teat microbiome analysis revealed reduced alpha diversity and significant changes in taxonomic profiles in quarters with a history of mastitis. Front Microbiol.

[28] Lima SF, Teixeira AG, Lima FS, Ganda EK, Higgins CH, Oikonomou G, Bicalho RC (2017). The bovine colostrum microbiome and its association with clinical mastitis. J Dairy Sci.

[29] Rupp R, Boichard D (2003). Genetics of resistance to mastitis in dairy cattle. Vet Res.

[30] Braem G, De Vliegher S, Verbist B, Heyndrickx M, Leroy F, De Vuyst L (2012). Culture-independent exploration of the teat apex microbiota of dairy cows reveals a wide bacterial species diversity. Vet Microbiol.

[31] Svennesen L, Nielsen SS, Mahmmod YS, Krömker V, Pedersen K, Klaas IC (2019). Association between teat skin colonization and intramammary infection with Staphylococcus aureus and Streptococcus agalactiae in herds with automatic milking systems. J Dairy Sci.

[32] Heikkilä A-M, Liski E, Pyörälä S, Taponen S (2018). Pathogen-specific production losses in bovine mastitis. J Dairy Sci.

[33] Østerås O, Solbu H, Refsdal A, Roalkvam T, Filseth O, Minsaas A (2007). Results and evaluation of thirty years of health recordings in the Norwegian dairy cattle population. J Dairy Sci.

[34] Callahan BJ, McMurdie PJ, Rosen MJ, Han AW, Johnson AJA, Holmes SP (2016). DADA2: high-resolution sample inference from Illumina amplicon data. Nat Methods.

[35] Murali A, Bhargava A, Wright ES (2018). IDTAXA: a novel approach for accurate taxonomic classification of microbiome sequences. Microbiome.

[36] Quast C, Pruesse E, Yilmaz P, Gerken J, Schweer T, Yarza P, Peplies J, Glöckner FO (2012). The SILVA ribosomal RNA gene database project: improved data processing and web-based tools. Nucleic Acids Res.

[37] Team RC. R: a language and environment for statistical computing. R Foundation for Statistical Computing, Vienna, Austria. 2020.

[38] Dixon P (2003). VEGAN, a package of R functions for community ecology. J Veg Sci.

[39] Lin H, Peddada SD (2020). Analysis of compositions of microbiomes with bias correction. Nat Commun.

