# Supplementary material for: Longitudinal dynamics of the bovine udder microbiota
Source: Anim Microbiome. 2022 Apr 8;4:26. doi: 10.1186/s42523-022-00177-w (PMC8994269; doi:10.1186/s42523-022-00177-w)
Supplement: Supplementary file 2 — Additional file 2. Day in milking for the 10 cows over the six samplings. [file 42523_2022_177_MOESM2_ESM.docx]

**Additional file 2**


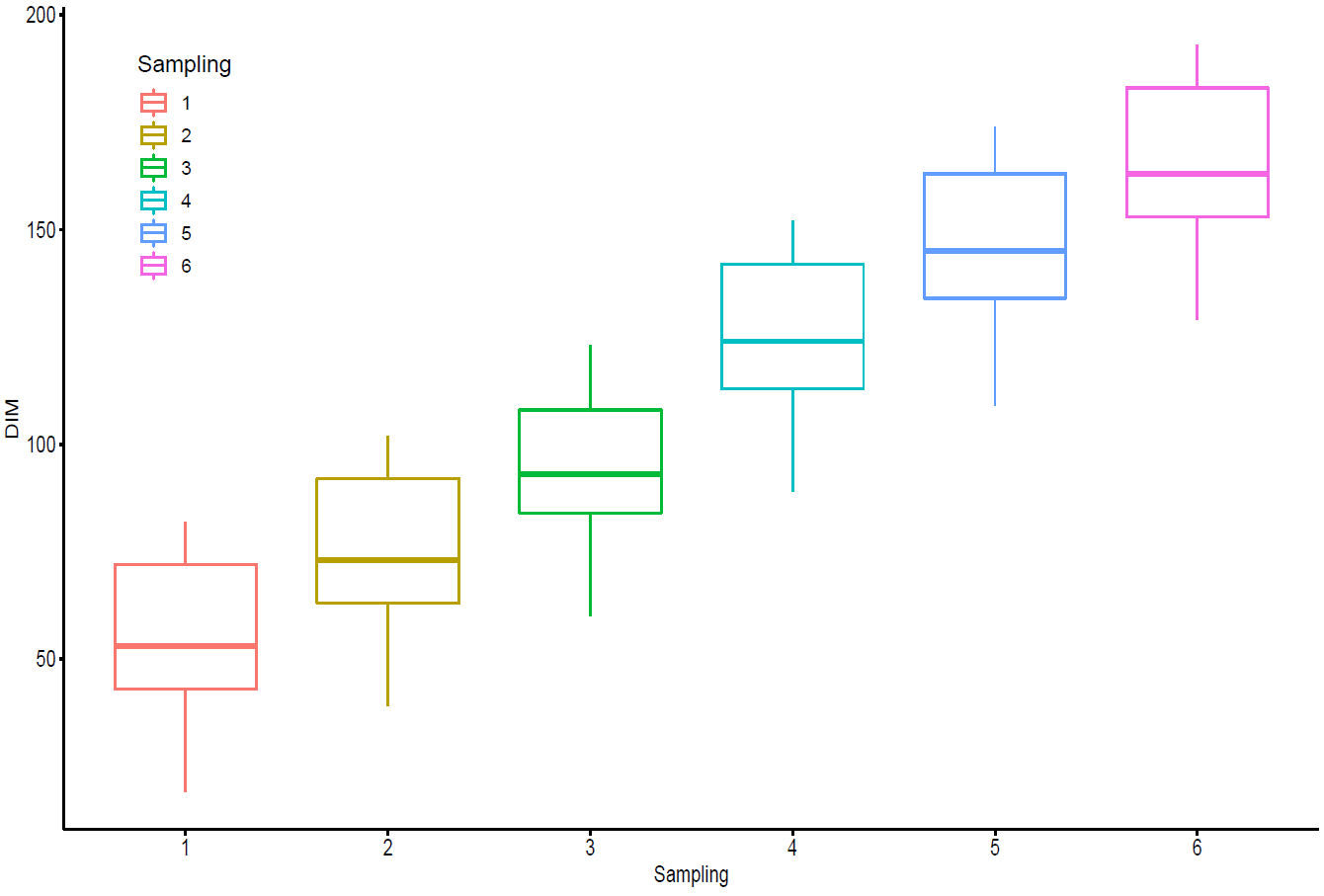


**Figure 1**. Day in milking (DIM) for the 10 cows over the six samplings. The quarter sampling occurred between day 19 and day 193 of the day in milking.
